# Circular RNAs in stem cells: from basic research to clinical implications

**DOI:** 10.1042/BSR20212510

**Published:** 2022-01-06

**Authors:** Hui-Juan Lu, Juan Li, Guodong Yang, Cun-Jian Yi, Daping Zhang, Fenggang Yu, Zhaowu Ma

**Affiliations:** 1 The First Affiliated Hospital of Yangtze University, Health Science Center, Yangtze University, Jingzhou, Hubei 434023, China; 2 School of Basic Medicine, Health Science Center, Yangtze University, 1 Nanhuan Road, Jingzhou, Hubei 434023, China; 3 Department of Obstetrics and Gynecology, The First Affiliated Hospital of Yangtze University, Jingzhou, Hubei 434023, China; 4 Key Laboratory of Environmental Health, Ministry of Education, Department of Toxicology, School of Public Health, Tongji Medical College, Huazhong University of Science and Technology, Wuhan, Hubei 430030, China; 5 Department of Oncology, Huanggang Central Hospital of Yangtze University, Huanggang, Hubei 438000, China; 6 Institute of Life Science, Yinfeng Biological Group, Jinan 250000, China

**Keywords:** circular RNAs, clinical implication, regulatory mechanisms, stem cells, stemness

## Abstract

Circular RNAs (circRNAs) are a special class of endogenous RNAs with a wide variety of pathophysiological functions via diverse mechanisms, including transcription, microRNA (miRNA) sponge, protein sponge/decoy, and translation. Stem cells are pluripotent cells with unique properties of self-renewal and differentiation. Dysregulated circRNAs identified in various stem cell types can affect stem cell self-renewal and differentiation potential by manipulating stemness. However, the emerging roles of circRNAs in stem cells remain largely unknown. This review summarizes the major functions and mechanisms of action of circRNAs in stem cell biology and disease progression. We also highlight circRNA-mediated common pathways in diverse stem cell types and discuss their diagnostic significance with respect to stem cell-based therapy.

## Introduction

Non-coding RNAs (ncRNAs), a class of endogenous RNAs, generally lack protein-coding potential [1]. Circular RNAs (circRNAs), a special class of ncRNAs, exert a widespread regulatory mechanism that orchestrates gene expression [2]. Increasing evidence suggests that circRNAs may regulate transcription, splicing, and chromatin interactions; function as microRNA (miRNA) decoys and sequester proteins; serve as protein scaffolds mediating the formation of complexes and template for translation and recruit proteins to specific locations [3,4]. Recently, several studies have reported that circRNAs play pivotal roles in the development and progression of various diseases, as well as in stem cell regulation [5,6]. Therefore, there remains a need for circRNA-based therapy for diverse diseases. Recently, circRNAs in stem cells has attracted considerable attention for their abundance in expression specificity, roles in promising clinical applications.

Stem cells are special types of undifferentiated cells that are capable of self-renewal as well as have the capacity to differentiate into different cell lineages [7,8]. There are two main types of stem cells, embryonic and adult [9]. Embryonic stem cells (ESCs) are pluripotent and, as a result, they can differentiate into all three germ layers [10]. Meanwhile, adult stem cells (ASCs) are multipotent with more limited potential to differentiate into different cell types, such as neural stem cells (NSCs), mesenchymal stem cells (MSCs), and hematopoietic stem cells (HSCs) [11]. Induced pluripotent stem (iPS) cells are a new type of pluripotent cells that can be obtained by reprogramming somatic cells into ES-like cells, which was heralded as a major breakthrough in stem cell research [12]. Accumulating studies have identified several signaling pathways involved in the self-renewal and differentiation of stem cells, including Wnt/β-catenin signaling and Notch signaling [13–15]. Currently, despite facing challenges, progress in the field of stem cells is very promising with reports of clinical success in treating various diseases such as neurodegenerative diseases and macular degeneration diseases [16].

Previous reviews have also summarized that circRNAs orchestrate the state and fate of stem cells, including different propensities for lineage selection upon differentiation, proliferation, and apoptosis [15,17,18]. However, to date, the functions and clinical applications of circRNAs in stem cells have not been systematically elucidated. This review focuses on elucidating the emerging roles of circRNAs in regulatory effects on stem cells, summarizing current progress of circRNAs in stem cells, and discussing their diagnostic potential and treatment implications.

## circRNAs as novel regulators in stem cell fate

circRNAs are generated by precursor mRNAs through non-canonical splicing, whereby the 3′ and 5′ ends of all or part of the linear mRNA molecule are covalently joined to form a continuous closed-loop [4,19]. The lack of 5′ caps and 3′ poly (A) tails make circRNAs resistant to RNase R and naturally more stable compared with linear RNAs [3]. circRNAs are highly enriched in eukaryotes, and many are evolutionarily conserved and expressed in a cell type/developmental stage/tissue-specific pattern [20,21]. With the advancement of novel technology and methods, circRNAs have been identified to be extremely abundant, relatively stable, diverse, prevalent, and conserved in different diseases [22]. Based on the source of the generation, circRNAs can be categorized into four types: exonic circRNAs, circular intronic circRNAs, exonic–intronic circRNAs, and circRNAs produced from tRNAs [4,23]. In addition, circRNAs may aggregate in cells to induce pathologies, such as developmental and degenerative disorders [24,25].

**Figure 1:**
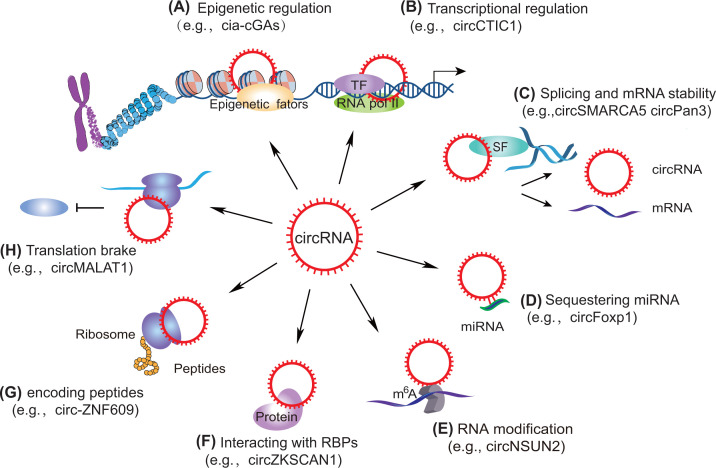
The expanding functions of circRNAs (**A**) CircRNAs can recruit epigenetic factors (e.g., cia-cGAs), (**B**) manipulate gene transcription by interacting with DNA sequences or transcription factors (e.g., circCTIC1), (**C**) regulate alternative splicing and affect mRNA stability (e.g., circSMARCA5 circPan3), (**D**) act as a sponge by binding to miRNA, (**E**) mediate RNA modification (e.g., circNSUN2), (**F**) interact with RBPs (e.g., circZKSCAN1), (**G**) encode functional peptides (e.g., circ-ZNF609), and (**H**) function as translation brake (e.g., circMALAT1).

Studies have indicated that circRNAs may take part in distinct physiological and pathological processes via diverse mechanisms, including the regulation of transcription or translation, miRNA sponge or protein sponge/decoy [3,26]. In addition, circRNAs are expressed in a tissue-specific pattern, suggesting that they act as promising diagnostic and therapeutic value in management of cancer and various diseases [27,28]. The mechanisms of circRNAs orchestrating gene expression can be summarized into the following aspects (Figure 1): (i) In the epigenetic layer, circRNAs recruit diverse epigenetic factors, including TET1 and DNMT1, to orchestrate the gene transcription and signal transduction [29]; (ii) In the transcriptional regulation, gene transcription is manipulated by circRNAs via interaction with transcriptional factors or cofactors [30]; (iii) At the post-transcriptional level, pre-mRNAs and mRNAs of many genes are modulated by circRNAs through competitively interacting with miRNAs, regulating transcription and alternative splicing, or interacting with RNA-binding proteins (RBPs) [2,31–33]; (iv) circRNAs orchestrate a broad repertoire of RNA modifications to affect their activation and stability [34–37]; (v) circRNAs could encode functional peptides that exert crucial roles in distinct biological processes [38]; (vi) circRNAs act as mRNA translation brake [39]. Taken together, the regulatory mechanisms of circRNAs in gene expression under various physiological and pathological conditions are very complicated and must be investigated in detail.

Stem cells are undifferentiated cells that can either self-renew or differentiate into specialized cells, mediating embryonic development, cellular differentiation, organ maintenance, tissue homeostasis, and wound repair [40]. Considering that stem cells have the potential to regenerate into diverse tissues and organs, stem cells may provide novel and effective treatment strategies against various diseases. Thus, scientists have paid attention to performing basic and biomedical research on stem cells for their unique properties in cell biology [41]. circRNAs are among many regulatory factors that affect the biological functions of stem cells. Numerous studies have revealed that circRNAs may participate in mediating the proliferation and differentiation of different types of stem cells and retrieving stemness [18,38]. However, current knowledge regarding the regulatory effects of circRNAs on stem cells at multiple levels, is still largely unknown [42].

Despite this, recent studies have demonstrated that circRNAs contribute to the self-renewal of stem cells as well as differentiation. Considering this concept, it is essential to determine how circRNAs influence the distinct types of stem cells, including MSCs and ESCs [18]. Current studies uncovered that circRNAs may play multiple roles in various types of stem cell (Table 1). These studies could bridge the mechanistic understanding of the effect of circRNAs on stemness-related diseases, thereby identifying novel targets of circRNA-related therapeutics and creating new strategies for improving stem cell therapy.

**Table 1:** The expanding roles of circRNAs in stem cells

Stem cells types	circRNAs	Interaction partners	Expression	Pathways	Functions	References
**Embryonic stem cells**						
ESCs	circBIRC6	miR-34a, miR-145			Attenuates the down-regulation of the target genes that maintain pluripotency and suppresses hESC differentiation	[43]
**Induced pluripotent stem cells**						
iPS cells (iPSCs)	circSLC8A1, circCACNA1D, circSPHKAP, and circALPK2				Overexpression of circSLC8A1 is related to heart disease	[44]
	circMYOD, circSLC8A1, circATXN7, and circPHF21A	Ribosome or Argonaute2 protein complexes			Bind to either the Argonaute2 protein complexes or ribosome	[45]
**Adult stem cells**						
HSCs	cia-cGAS		Up		Prevents long-term HSCs from failure	[46]
Adipose-derived stem cells	circRFWD2 and circINO80	hsa-miR-6817-5p	Up		Impacts osteogenic differentiation of hASCs induced by recombinant NELL-1	[47]
	circPOMT1 and circMCM3AP	hsa-miR-6881-3p	Down	BMPs signaling pathway	Might influence the osteogenic differentiation of human hASCs	[48]
	hsa_circH19				Knockout of hsa_circH19 facilitates the adipogenic differentiation of hADCSs by targeting PTBP1	[49]
NSCs	circRNA (rat_circ:chr15: 9915223-9915671)	miR-138-5p			Binds to miR-138-5p as a latent inhibitory regulator in NSCs proliferation	[50]
	circHIPK2, circTLK1				Circhipk2 can reduce the fate of neurons in the process of NSC differentiation; the silencing of circTLK1 is good for the infarct volume and functional recovery from stroke	[51]
	CircHIPK2	miR-124	Up		Inhibits NSCs to differentiate into neurons and hindered the plasticity of neurons to OGD/R	[52]
	circTLK1	miR-335-3p	Up		Strengthens neurological deficit and neuronal damage after ischemic stroke through miR-335-3p/TIPARP	[53]
Intestinal stem cells (ISCs)	circPan3		Up		In Lgr5 ISCs, knockdown circPan3 diminishes the regeneration of gut epithelium and their self-renewal ability	[54]
Epidermal stem cells (EpSCs)	circZNF91	miR-23b-3p	Up		Implicates in differentiation of EpSCs to keratinocytes	[55]
Maxillary sinus membrane stem cells (MSMSCs)	circRNA_33287	miR-214-3p	Up	circRNA_33287/miR-214-3p/Runx3 pathway	Regulates the osteoblastic differentiation of MSMSCs	[56]
Myoblast cells	circ-FoxO3	miR-138-5p			Inhibits myoblast cells differentiation	[57]
Skeletal muscle satellite cells	CDR1as	miR-7		CDR1as/miR-7/IGF1R regulatory pathway	Facilitates skeletal muscle satellite cell myogenic differentiation	[58]
**Mesenchymal stem cells**						
	circFOXP1	miR-17-3p/miR-127-5p		Non-canonical Wnt and EGFR	Silencing of circFOXP1 dramatically impairs MSC differentiation	[59]
	Hsa_circ_0074834	miR-942-5p	Down		Promotes the repair of bone defects and osteogenic differentiation of BMSCs	[60]
	circRNA_014511	miR-29b-2-5p			Inhibits the expression of P53 and decreases the radio sensitivity of BMSCs	[61]
	CDR1as	miR-7-5p	Up	CDR1as-miR-7-5p- WNT5B axis	Inhibits osteogenic and facilitates adipogenic differentiation of BMSCs	[62]
	circRNA_0006393	miR-145-5p		miR-145-5p/FOXO1 pathway	Promotes osteogenesis	[63]
	mmu_circRNA_ 003795	miR-504-3p			Orchestrates the expression of FOSL2 by sequestering miR-504-3p, leading to alterations in BMSC proliferation	[64]
	hsa_circ_0032599, hsa_circ_0032600 and hsa_circ_0032601		Up		Promotes the osteogenic differentiation of hBMSCs on titanium by surface mechanical attrition treatment (SMAT)	[65]
	circRNA 0020	miRNA-206-3p	Up	circRNA 0020/miR-206-3p/Nnmt axis	Participates in bone formation and osteogenic differentiation of stem cells	[66]
	circRNA 3832	miR-3473e	Up	circRNA 3832/miR-3473e/Runx3 axis	Involved in bone formation and osteogenic differentiation of stem cells	[66]
	mmu-circRNA_ 016901	miRNA1249-5p		TGF-β3	Participates in the regulation of the radiation damage mechanism of BMSCs	[67]
MSC: human periodontal ligament stem cells	circRNA3140	miRNA-21			Regulates miRNAs-mediated osteogenic differentiation in MSCs	[68]
	CDR1as	miR-7		ERK	Mediates the inhibition effect of LPS on cell proliferation	[69]
	CDR1as	miR-7	Up	MiR-7-5p/GDF5/SMAD and p38MAPK signal pathway	Promotes osteogenic differentiation of PDLSCs	[70]
Human umbilical cord MSCs	CDR1as		Up		Regulates the proliferation and differentiation of hucMSCs	[71]
	circHIPK3	miR-421		Exosome/circHIPK3/FOXO3a pathway	Repairs ischemic injury	[72]
Adipose-derived mesenchymal stem cells (ADSCs)	mmu_circ_0000623	miR-125	Down		Exosomes from ADSCs modified by mmu_circ_0000623 impede liver fibrosis via promoting autophagy	[73]
	mmu_circ_0000250	miR-128-3p			Exosomes derived from adipose-derived MSCs modified by mmu_circ_0000250 facilitate wound healing via inducing autophagy mediated by miR-128-3p/SIRT1	[74]
**Cancer stem cells**						
Cancer stem cells (CSCs)	CircCTIC1	NURF complex	Up		Drives the self-renewal of colon TICs	[75]
	CircZKSCAN1	FMRP		Qki5-circZKSCAN1-FMRP-CCAR1-Wnt signaling axis	Interacts with FMRP to block FMRP binding to CCAR1 mRNA, resulting in inhibit Wnt signaling and negatively modulates CSCs	[76]
	CircGprc5a		Up	CircGprc5a-peptide-Gprc5a	CircGpr5a knockout diminishes the metastasis and self-renewal of bladder CSCs	[78]
	CircPRMT5	miR-30c	Up	circPRMT5/miR-30c/SNAIL1/E-cadherin pathway	Promotes UCB cell’s EMT and/or aggressiveness	[79]
	Circ008913	miR-889			Promotes the expression of ZEB1 and the cell-surface markers of skin stem cells, and facilitates tumorigenesis by sponging miR-889	[80]
	CircZEB1	hsa-mir200a-3p and hsa-mir141-3p			Modulates miRNA-mediated circuits and affects melanoma plasticity	[81]
	CircPTN	miR-145-5p/miR-330-5p			Facilitates self-renewal and up-regulated stemness markers (SOX2, Nestin, SOX9, and CD133)	[82]
	mmu_circ_0000730	mmu-miR-466i-3p			Hinders many EMT-related genes, including SOX9 and stemness of CSCs	[83]
	CircRNA_103809	miR-511	Up		Facilitates the migration, invasion and self-renewal, abilities of bladder cancer	[84]
	Cir-CCDC66		Up	Hepatocyte growth factor/c-Met pathway	Enhances the enrichment of CSCs	[85]
	Circ-NOTCH1	miR-449c-5p	Up	miR-449c-5p/MYC/NOTCH1 axis	Facilitates stemness and metastasis in gastric cancer (GC)	[86]

Abbreviations: CDR1as, cerebellar degeneration-related protein 1 transcript; ERK, extracellular signal-regulated kinase; FOSL2, FOS-like 2 AP-1 transcription factor subunit; hASC, human adipose-derived stem cell; hESC, human embryonic stem cell; hucMSC, human umbilical cord-derived MSC; LPS, lipopolysaccharide; NELL-1, Nel-like molecule, type 1; OGD/R, oxygen-glucose deprivation/reperfusion; PDLSC, lipopolysaccharide-treated periodontal ligament stem cell; UCB, urothelial carcinoma of the bladder.

## Functions and mechanisms of circRNAs in stem cells

### circRNAs in ESCs

ESCs are pluripotent, self-renewing cells that are derived from the inner cell mass of the developing blastocyst [87]. ESCs can grow indefinitely while maintaining pluripotency [42,88–90]. ESCs can be cultivated for extended periods and genetically manipulated without the loss of their pluripotency. Particularly, ESCs have a remarkable ability to preserve transcriptional patterns via mitosis, relying on a network of sequence-specific transcription factors [91].

**Figure 2:**
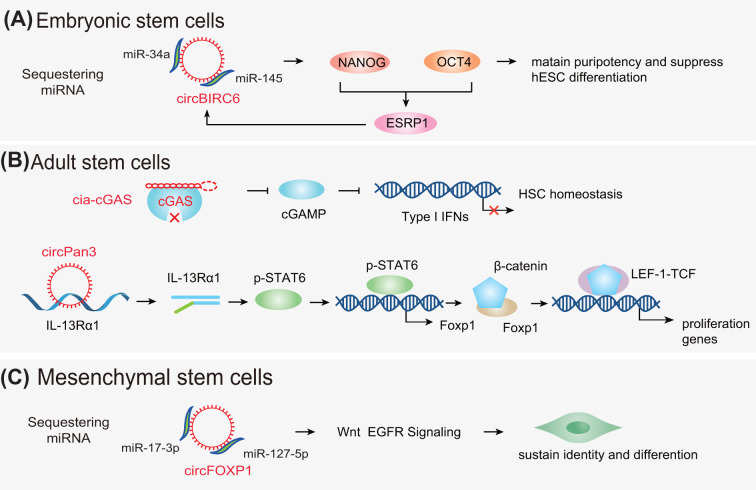
The mechanisms of action of circRNAs in stem cells (**A**) In ESCs, circRNAs (e.g., circBIRC6) regulate the generation of stemness-related genes (e.g., OCT4 and NANOG). (**B**) In ASCs, cia-cGAS binds to cGAS to impede cGAMP, resulting in suppression of type I interferons (IFNs) transcription. circRNAs (e.g., circPan3) affect the stability of mRNA. (**C**) In MSCs, circRNAs (e.g., circFOXP1) sequester miRNAs through Wnt, EGFR signaling pathways. Abbreviations: cGAMP, cyclic GMP-AMP; cGAS, cGAMP synthase; EGFR, epidermal growth factor receptor.

Studies have demonstrated that circRNAs exert significant and comprehensive effects on ESCs, including modulation of human pluripotency and differentiation. For instance, one study has reported that circBIRC6 is up-regulated in undifferentiated human embryonic stem cells (hESCs) and positively associated with the pluripotent state. Moreover, circBIRC6 was found to be enriched in the AGO_2_ complex and served as the binding platform of miR-145 and miR-34a, promoting the target genes that maintain pluripotency and suppress hESC differentiation. The authors further verified that in hESCs, circBIRC6 expression was facilitated by the splicing factor, ESRP1, whose biogenesis was modulated by OCT4 and NANOG, the core pluripotency-associated factors. Thus, the present study indicated that the circRNA could sponge miRNA to modulate the molecular circuitry, orchestrating human differentiation and pluripotency (Figure 2A) [43]. Lorenzo et al. had identified the expression profiles of circRNAs in mouse ESC-derived motor neurons and found that the generation of these circRNAs was manipulated by RBP FUS [92]. To explore the genome-wide circRNA profile in the heart, Tan et al. conducted deep RNA-sequencing on ribosomal-depleted RNA obtained from human ESC-derived cardiomyocytes. The results showed that circSLC8A1-1 was an abundant cardiac-expressed circRNA [93]. Current findings revealed that circRNAs could interact with miRNAs, resulting in the attenuation of the down-regulation of target genes and mediation of hESC differentiation, thus providing the foundation for further exploration of the roles of circRNAs epigenetic regulation patterns in ESCs. Nevertheless, more studies on the functions of circRNAs in ESCs are warranted to further explore the function and potential effects of circRNAs on ESCs.

### circRNAs in iPS cells

iPS cells, are a type of pluripotent stem cell (PSC) derived from adult somatic cells that have been genetically reprogrammed to an embryonic stem (ES) cell-like state through the forced expression of Yamanaka factors: Klf4, c-Myc, Sox2, and Oct3/4 [90].

The process of converting lineage-committed cells into iPS cells is called reprogramming [41]. Somatic cells can be reprogrammed into iPS cells by transient ectopic up-regulation of the stemness-related transcription factors [94]. iPS cells have tremendous therapeutic potential, owing to their ability to reprogram patient somatic cells [95]. Human iPS (hiPS) cells offer a powerful platform for disease modeling and have uncovered new probabilities for understanding the mechanisms controlling human physiology, genetics, and biology [96]. NcRNAs, including circRNAs, are now universally acknowledged as a significant class of modulators of the pluripotent state and lineage commitment [95]. For instance, in the hiPS cell-derived cardiomyocyte model, the expression of numerous circRNAs such as circSLC8A1, circPHF21A, circMYOD, and circATXN7, which bind to not only ribosome but also the Argonaute2 protein complexes, showed significant changes. Thus, this model discloses novel features of potential disease-related circRNAs, which may become novel therapeutic targets [45]. Another group found that hiPS cell derived from fibroblasts and further differentiated to cardiomyocyte through the regulation of WNT signaling. circRNAs are highly expressed in cardiomyocytes derived from differentiated hiPS cells, and thus heart-specific circRNAs, including circALPK2, circCACNA1D, circSLC8A1 and circSPHKAP, may function as biomarkers of cardiomyocytes. Furthermore, the expression of circSLC8A1 was excessive in dilated cardiomyopathy patients’ cardiac tissues [44]. Another study on the circRNA expression profiles of hiPS cell-derived cardiomyocytes indicated that circSLC8A1 might sponge miR-133a in cardiac myocytes through being associated with ribosomes, and flagged as m6A-methylated [97]. Consequently, the overexpression of circSLC8A1 could be an indication of pathological status in heart disease. CircITCH down-regulated in doxorubicin-treated hiPS cell-derived cardiomyocytes can up-regulate the expression of SIRT6, Survivin, and SERCA2a to alleviate DOX-induced cardiomyocyte injury caused by sponging miR-330-5p [98].

Collectively, the use of iPS cell-based disease models, including hiPS cell-derived cardiomyocytes, hematological, neurodegenerative, and familial syndromes, hold great promise for understanding disease mechanisms and accelerating discovery of effective treatments [99,100]. A better understanding in circRNAs in the context of iPS cells would certainly benefit future biomedical research aiming to utilize iPS cells in the clinic. However, iPSC-based therapeutic approaches such as drug discovery and cell replacement therapy remain in their infancy, and more research work needs to be done to improve our understanding on potential of iPS cells.

### circRNAs in ASCs

ASCs can undergo differentiation and generate multiple lineages as well as simultaneously self-renew [101]. Based on the tissue source, ASCs can be grouped into several subtypes, including HSCs, intestinal stem cells (ISCs), NSCs, endothelial stem cells, and cardiac stem cells, etc. [102].

#### circRNAs in HSCs

HSCs are a rare population of cells residing in the bone marrow and are capable of generating an entire hematopoietic system [103]. HSC-based cell transplantation is a curative therapy for several hematologic malignancies and other disorders [104]. Accumulating studies have demonstrated that circRNAs may influence HSC homeostasis and differentiation potentials. For example, a circRNA derived from D430042O09Rik gene transcripts, termed as cia-cGAS (circular RNA antagonist for cGAS), was up-regulated in long-term HSCs (LT-HSCs), promoted the number of dormant LT-HSCs in mice, and suppressed the expression of type I interferons (IFNs) in the bone marrow. Under a homeostatic state, cia-cGAS alleviated DNA sensor cyclic GMP-AMP (cGAMP) synthase (cGAS) synthesis, which led to suppression of cGAS-induced production of type I IFNs in LT-HSCs and prevented dormant LT-HSCs from cGAS-induced exhaustion. These findings unveiled a mechanism by which cia-cGAS suppressed nuclear cGAS by inhibiting its enzymatic activity and preventing cGAS from recognizing self-DNA to keep host homeostasis (Figure 2B) [46]. Jadhav et al. identified circASXL1-1 and circASXL1-2 from the SXL1 gene locus in the THP-1 leukemic cell line, which were also expressed in various cell lines. Furthermore, this study suggested that circASXL1-1 possibly changed the activity of the PR-BUB complex by physically associating with BAP1 and might influence the differentiation of HSCs in the myeloid lineage [105]. Overall, HSCs have shown promising clinical applications, targeting circRNAs of HSCs may contribute to a better treatment of hematologic diseases.

#### circRNAs in adipose-derived stem cells

Adipose-derived stem cells (ADSCs) can self-renew and differentiate along multiple cell lineages [106]. Accumulating studies have demonstrated the potentials of ADSCs for stem cells therapy for diverse clinical disorders. Thus, ADSCs are one of the most widely used types of stem cells in clinical settings due to their ability of high rate of proliferation, anti-fibrotic, anti-apoptotic, and anti-inflammation [107]. circRNAs have emerged as crucial regulators in ADSCs in a multitude of phenotypes, ranging from adipogenic differentiation to osteogenesis. For example, overexpressed hsa_circH19 has been demonstrated as an independent risk factor for metabolic syndrome, which may sequester polypyrimidine tract-binding protein 1 (PTBP1), resulting in the inhibition of sterol-regulatory element-binding proteins (SREBP1s) precursor cleavage. Mechanistically, depletion of hsa_circH19 induces translocation of SREBP1 from the cytoplasm to the nucleus, in the presence of PTBP1, thereby significantly up-regulating the expression of adipogenic genes in ADSCs and promoting the formation of lipid droplets [49]. Similarly, circRFWD2 and circINO80 were reported to be overexpressed in recombinant Nel-like molecule, type 1 (NELL-1)-mediated osteogenesis. Knockdown of circRFWD2 exerted the positive role of NELL-1 in osteogenic differentiation of human adipose-derived stem cells (hASCs) via sponging hsa-miR-6817-5p, which emphasized circRFWD2 as a promising molecular target for the regulation of osteogenesis and bone regeneration [47]. circRNA-23525 was overexpressed and up-regulated the expression of Runx2, alkaline phosphatase (ALP), and OCN by sponging miR-30a-3p, leading to osteoblastic differentiation of adipose-derived MSCs [108]. circSIPA1L1 up-regulated ALPL and promoted osteogenic differentiation of stem cells from apical papilla via targeting miR-204-5p [109]. The expression of circPOMT1 and circMCM3AP was low during the osteogenesis of hASCs. Mechanistically, circPOMT1 and circMCM3AP may control the osteogenic differentiation of hASCs by interacting with hsa-miR-6881-3p and targeting Smad6 and Chordin, the two critical inhibitors of the bone morphogenetic proteins (BMPs) signaling pathway. CircPOMT1 and circMCM3AP may act as potential targets for the repair of bone defects [48]. In a mouse model, exosomes derived from circAkap7-modified ADSCs, namely exosomal circAkap7, prevented cerebral ischemic injury via facilitating autophagy and alleviating oxidative stress [110]. Taken together, these studies provide novel insights into the altered and specific circRNAs in ADSCs via binding to proteins or miRNAs, thus affecting adipose and bone differentiation, along with lipid metabolism and osteogenesis.

#### circRNAs in NSCs

NSCs are another type of ASCs identified and used for cell therapy in neurological diseases [111]. NSCs derived from the mammalian brain are the origins of glia and new neurons contributing to complex sensory and cognitive functions and generate all the differentiated neural cells of the mammalian central nervous system via the formation of intermediate precursors [112,113]. Exploring the functional integration of newborn neurons or the modulatory mechanisms of stem cells neurogenesis provides the basis for grafted stem cell treatment for diseases such as Alzheimer’s disease [114]. Clinical trials have been conducted for the usage of fetal NSCs against distinct neurological disorders [111]. Recent findings revealed that many circRNAs contribute to the regulation of NSCs function. For example, Cao et al. demonstrated that silencing circRNA cZNF292 alleviates oxygen-glucose deprivation/reperfusion (OGD/R)-induced NSC injury, thus promoting the activation of protein kinase C/extracellular signal-regulated kinase and Wnt/β-catenin pathways by up-regulating miR-22 [115]. In another study, it was observed that during the differentiation of NSCs, the expression of circHIPK2 was decreased, which played a neuroprotective role and facilitated nerve recovery after stroke. This study demonstrated that circHIPK2/Smox/TUJ1 axis regulated NSC neuronal plasticity against OGD/R and silencing of circHIPK2 facilitated NSCs directionally differentiated to neurons [52]. In a focal cerebral ischemia and reperfusion model, circTLK1 was significantly up-regulated in the brain tissues and interacted with miR-335-3p to enhance ADP-ribose polymerase, thus aggravated neuronal injury [53]. Recent studies have identified the profile of circRNAs in different models, such as during mouse NSC differentiation and in the subventricular zone of rat adult NSCs [50,116]. Therefore, a cell therapy approach such as transplantation of NSC in combination with the silencing of circRNA within the transplanted cells could serve as an attractive method to regulate NSC function, thus favoring brain regeneration and promoting functional recovery from cerebral ischemia [51]. Together, circRNAs could modulate NSC function to support brain regeneration and facilitate functional recovery from cerebral ischemia and may serve as potential targets for the treatment of neurological diseases.

#### circRNAs in ISCs

ISCs are highly proliferative cells that fuel the continuous renewal of the intestinal epithelium, and could generate daughter or progenitor cells, which can subsequently differentiate into the mature cell types required for normal gut function [117,118]. ISCs can be identified based on the expression of a unique marker gene, namely Lgr5 [119]. Lgr5^+^ ISCs sustain renewal and reside between terminally differentiated Paneth cells at the bottom of the intestinal crypt. Sato et al. demonstrated that Lgr5^+^ ISCs could be cultivated to build epithelial structures that represent hallmarks of the *in vivo* epithelium [120]. ISCs are retained via stemness signaling pathways for precise regulation of differentiation and self-renewal under homeostasis. CircPan3 (originating from the Pan3 gene transcript) is up-regulated in human and mouse ISCs and drives the self-renewal of ISCs. CircPan3 directly interacts with mRNA encoding IL-13Rα1 (the cytokine IL-13 receptor subunit) in ISCs to increase its stability, leading to IL-13Rα1 expression in ISCs. IL-13 is generated by group 2 innate lymphoid cells in the crypt niche engaged IL-13Rα1 on crypt ISCs, and can activate the IL-13‒IL-13R-mediated signaling pathway, which in turn acts as a trigger for the expression of the transcription factor, Foxp1. Foxp1 directly binds to β-catenin via rendering its nuclear translocation, which activates the β-catenin pathway and maintains Lgr5^+^ ISCs. Collectively, in Lgr5-GFP^+^ ISCs, circPan3 facilitates ISC self-renewal ability via crypt niche ILC2-induced IL-1‒IL-13R signaling pathway (Figure 2B) [54]. circRNAs play critical roles in various biological processes, however, their roles in ISCs self-renewal remain elusive, more research on the role of circRNAs in ISCs needs to be conducted in the near future.

#### circRNAs in MSCs

MSCs (or mesenchymal stromal cells) possess self-renewal abilities and multilineage differentiation potential into mesodermal lineage, including adipocytes, osteocytes, and chondrocytes. They exist in various tissues, including bone marrow, adipose tissue, umbilical cord, and dental pulp [121,122]. Due to their chemotactic property, they have been commonly utilized to treat multiple diseases, including cancer drug resistance, tissue injury and regeneration, and aging, through diverse mechanisms involving angiogenesis and stemness promotion [123–126].

Beside regulating the proliferation and differentiation of MSCs, circRNAs also appear to play roles in diverse phenotypes of MSCs, such as angiogenesis and osteogenesis [60,62,63,70,127]. For instance, Cherubini et al*.* first identified that circFOXP1 was highly expressed in MSCs and promoted MSC’s proliferation and differentiation in culture and *in vivo* by binding to miR-127-5p and miR-17-3p in stem cell fate deciding processes. CircFOXP1 contributed to the regulation of epidermal growth factor receptor (EGFR) and non-canonical Wnt pathways in the maintenance of MSC identity and manipulation of differentiation (Figure 2C) [59]. Cerebellar degeneration-related protein 1 transcript (CDR1as) was up-regulated in human umbilical cord-derived MSCs (hucMSCs). CDR1as knockdown induced hucMSC differentiation potential impairment, cell cycle arrest, and cell apoptosis, thereby providing the foundation for MSC modification and stem cell therapy, and tissue regeneration [71]. In contrast, the expression of CDR1as was significantly low in lipopolysaccharide (LPS)-treated periodontal ligament stem cells (PDLSCs, characterized as MSCs), which facilitated LPS-induced proliferative inhibition of PDLSCs. Mechanistically, CDR1as/ciRS-7 sequesters miR-7 to modulate the suppression effect of LPS on cell proliferation via activating the extracellular signal-regulated kinase (ERK) signal pathway [21]. Another study revealed the molecular mechanisms of CDR1as/miR-7 on PDLSCs proliferation ability within their surrounding inflammatory microenvironment of periodontitis [69]. circRNAs in MSCs play pivotal functions and have the potential to be used as biomarkers and therapeutic targets. A recent study found that the circRNAs are differentially expressed in hUCMSCs. This finding may establish the foundation to reveal the mechanism underlying the differentiation of hUCMSCs into cardiomyogenically induced cells and offer clues for MSC transplantation treatment [128]. Wang et al. showed that circRNA3140 caused MF-induced osteogenic differentiation of PDLSCs, which may contribute to the alveolar bone remodeling and the orthodontic tooth movement process [68]. Human umbilical cord mesenchymal stem cell-derived exosomes (UMSC-Exo) carrying circHIPK3 could inhibit inflammation and restore ischemic muscle injury via miR-421/FOXO3a axis [72]. Some of the identified circRNAs could act as important regulators to impact stem-related phenotypes. A study revealed that the PDLSC-derived exosomal circRNA expression profile was significant altered during the initial stage of osteogenic differentiation of PDLSCs [129]. Another study revealed different circRNAs in the pathogenesis of psoriasis, thus providing novel prospective biomarkers for the diagnosis and prognosis of psoriasis pathogenesis [130].

Recently, it has been reported that several circRNAs could promote the angiogenesis‒osteogenesis coupling process in bone MSCs (BMSCs) by sponging miRNAs. For instance, hsa_circ_0074834 could facilitate osteogenic differentiation of BMSCs and the repair of bone defects through miR-942-5p/VEGF/ZEB1 axis [60]. circRNA_014511 was identified in mouse bone marrow MSCs (BMMSCs) and reportedly suppresses p53 expression by acting as a sponge of miR-29b-2-5p and diminish the radiosensitivity of BMMSCs by infecting cell apoptosis and cell cycle [61]. CDR1as was markedly overexpressed during the osteogenic differentiation, facilitating the Alizarin Red staining, ALP activity, and osteogenic differentiation of PDLSCs. This study uncovered new mechanisms involving CDR1as/miR-7/GDF5 (growth differentiation factor 5)/p38 mitogen-activated protein kinases/Smad1/5/8 axis underlying osteogenic differentiation, suggesting the potential of CDR1 as a therapeutic target in periodontal tissue and bone regeneration [70]. In adipogenic/osteogenic differentiation disorder, CDR1as up-regulated the expression of WNT5B by sponging miR-7-5p, thereby increasing adipogenic differentiation and decreasing osteogenic differentiation of BMSCs [62]. Wang et al. identified a circRNA hsa_circ_0006393 in glucocorticoid-induced osteoporosis, localized in the nucleus and cytoplasm of BMSCs. Further functional assays revealed that hsa_circ_0006393 regulated bone metabolism modulation via the miR-145-5p/FOXO1 signaling axis [63]. The expression of hsa_circ_0004588, hsa_circ_0005936, and hsa_circ_0000219 was significantly low in the BMSCs of patients with osteonecrosis of the femoral head (ONFH). These circRNAs can modulate the progression of ONFH by regulating the differentiation and proliferation of BMSCs via sponging distinct miRNAs. This study was the first to report the dynamic expression and role of circRNAs in patients diagnosed with ONFH [127]. In the calcitonin gene-related peptide (CGRP)-stimulated BMSCs of mice model, CGRP sequestered miR-504-3p via mmu_circRNA_003795, leading to up-regulation of FOS like 2 AP-1 transcription factor subunit (FOSL2) and facilitating the proliferation of BMSCs by influencing the cell cycle [64].

Certain circRNAs have been shown to play a significant role in the modulation of MSCs phenotypes (e.g., angiogenesis and osteonecrosis). These findings suggest that targeting circRNAs may offer novel insights into regeneration and wound repair.

#### circRNAs in other types of ASCs

circRNAs can serve as multifunctional mediators to regulate other types of ASCs. For example, circZNF91 affects keratinocyte differentiation in epidermal stem cells (EpSCs) [55]. circRNA_33287 regulated the osteoblastic differentiation of maxillary sinus membrane stem cells (MSMSCs) [56]. In addition, a recent study revealed that cerebellar degeneration-related protein 1 transcript (CDR1as) facilitated the myogenic differentiation of skeletal muscle satellite cells [58]. Recent studies have also identified several circRNAs in spermatogonial stem cells (SSCs), and circ-FoxO3 could modulate muscle differentiation [57,131]. Although the current studies on circRNAs and other types of ASCs have emerged, these studies do not clearly explain how circRNAs play a role in these cells, and the specific molecular mechanism is still unknown.

Several studies have been reported on the roles of circRNAs in other stem cells (e.g., EpSC and SSCs). circRNAs possess vital roles in various ASCs with diverse mechanisms. ASCs are expected to replace bone marrow cells as important materials for regenerative cell therapy in the near future. Considering a large amount of circRNAs identified, we still have a long way to understand the full range of roles of these regulators in ASCs.

### circRNAs in cancer stem cells

Cancer stem cells (CSCs) represent a fraction of undifferentiated cancer cells that exhibit stem cell-like features, are able to give rise to a whole new tumor on its own, as compared with other cancer cells that lack this ability, and could generate clones of continuously growing tumors [132,133]. Several theories have been proposed to explain the potential origins of CSCs, including the embryonal-rest hypothesis, CSC hypothesis, and origin of CSCs [134–138]. CSCs have been identified in a series of solid tumors, such as lung CSCs, ovarian CSCs, breast CSCs, or hepatocellular CSCs [139–141]. Extensive studies have shown that CSCs play a vital role in tumor initiation, maintenance, and metastasis, contributing to conventional therapy failure and cancer recurrence because of their treatment resistance and self-regeneration characteristics [142–145].

**Figure 3:**
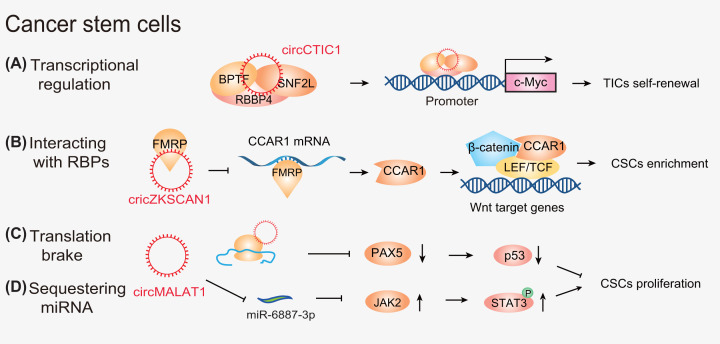
The mechanisms of action of circRNAs in CSCs CircRNAs (**A**) act as scaffolds (e.g., circCTIC1), (**B**) harness RNA–protein interactions. In adition, circRNAs (e.g., circMALAT1) (C) function as mRNA translation brake and (D) act as miRNA (e.g., circMALAT1) sponges.

Mounting evidence has shown that circRNAs can function as potential diagnostic and prognostic biomarkers, and as therapeutic targets in multiple cancer types through their interactions with CSCs [146]. Recent studies have uncovered a link between circRNAs and CSCs through diverse regulatory mechanisms involving in gene transcription and translation. For example, circCTIC1 was elevated in colon tumor-initiating cells (TICs) and colon cancer and drove colon tumorigenesis and TIC self-renewal. Mechanistically, circCTIC1 facilitated the transcriptional initiation of c-Myc via recruitment of the nuclear remodeling factor complex on to the promoter of c-Myc (Figure 3A) [75]. Intriguingly, some circRNAs also act on the processing of mRNAs to influence their stabilities. For example, circZKSCAN1 was down-regulated in hepatocellular carcinoma (HCC) patient-derived xenograft mouse models and was associated with overall survival and recurrence-free survival. In HCC CSCs, circZKSCAN1 modulated the RBP FMRP (fragile X mental retardation protein) and CCAR1 (cell cycle and apoptosis regulator 1) to activate the Wnt/β-catenin pathway, and thus impeded cell stemness. Moreover, the expression of RNA-splicing protein Qkit5 (Quaking 5) was low in HCC tissues and was related to the decline in circZKSCAN1. This study suggested a potential therapeutic target of the newly discovered Qki5-circZKSCAN1-FMRP-CCAR1-Wnt axis in HCC treatment (Figure 3B) [76]. Collectively, these findings suggest that circRNAs may exert a wide variety of roles to impact tumor progression and anti-cancer drug resistance by modulating the expression of many genes of stemness.

It has also been reported that circRNAs can encode certain functional peptides by interacting with a variety of biomolecules. For example, circGprc5a was highly expressed in bladder tumors and bladder CSCs and drove the self-renewal and metastasis of bladder CSCs. Therefore, the circGprc5a-peptide-Gprc5a axis could be a promising diagnostic and therapeutic target for bladder cancer [78].

Intriguingly, some circRNAs have been reported to manipulate the ability of CSCs via interconnected molecular mechanisms; for instance, circ-MALAT1 is highly expressed in CSCs from clinical HCC samples. Mechanistically, circ-MALAT1 can not only inhibit PAX5 mRNA translation by mRNA braking (Figure 3C), but also sequester miR-6887-3p to activate the JAK2/STAT3 signaling pathway, contributing to self-renewal of hepatocellular CSCs (Figure 3D) [39].

circRNAs can act as miRNA sponges, an extensive mechanism to manipulate a series of signaling cascades. In an arsenite-induced stem cell-like model in T-HaCaT cells, circ008913, derived from ADAT1, was down-regulated and impacted colony formation, invasion, migration, and tumor progression. Functional assays revealed that circ008913 sponges miR-889 and down-regulates its target DAB2IP, resulting in ZEB1 up-regulation, and is involved in the arsenite-induced acquisition of CSC-like properties that lead to malignant transformation [80]. Specifically, in primary human melanoma cells, circZEB1 was highly expressed to infect the entire phenotypic transformation via influencing the miR-141-3p/miR-200a-3p/ZEB1 axis, which allowed the tumor to tightly control the number of CSCs present in the population [81]. Hsa_circ_0082096, derived from ZNF800, and hsa_circ_0066631, derived from DCBLD2, were dramatically overexpressed in CSC-enriched CRC spheroid cells. Furthermore, the study showed that the novel circRNA–miRNA–mRNA axis participated in several signaling pathways in manipulating CSC stemness, including Wnt/β-catenin [147].

Successive generations of xenografts yielded a highly malignant EBV-associated gastric cancer (EBVaGC) cell line, SNU-4th, which displays typical CSC properties. In EBVaGC, ebv-circLMP2A, an EBV-encoded circRNA, was up-regulated and related to metastasis and poor prognosis in patients diagnosed with EBVaGC, thereby keeping stemness phenotypes by targeting miR-3908/TRIM59/p53 signaling axis [148]. Moreover, overexpression of circPTN facilitated self-renewal and up-regulated stemness markers (such as CD133) by binding to miR-330-5p/miR-145-5p, facilitating proliferation and up-regulating ITGA5/SOX9 in glioma stem cells [82]. In bladder CSCs, circRNA-103809 was highly expressed and promoted the self-renewal, migration, and invasion capabilities of bladder cancer via sponging miR-511 [84]. Cir-CCDC66 was overexpressed in CSCs spheres and renal carcinoma cancer cell lines to enhance the CSC enrichment through hepatocyte growth factor/c-Met signaling pathway [85]. These studies provide evidence that circRNAs mainly function as miRNA sponges, which suggests that the circRNA–miRNA–mRNA regulatory axis may be pivotal for CSCs self-renewal or tumor progression.

Mounting evidence has uncovered that CSCs may contribute to tumor growth, cancer recurrence, and therapy resistance [149,150]. Moreover, several reports have suggested that circRNAs are also deeply involved in resistance to anti-cancer drugs, and the vital role of circRNAs in mediating anti-cancer drug resistance is emerging [22,151]. Targeting CSCs could be a promising and novel strategy to circumvent CSC-mediating drug resistance, thereby decreasing treatment failure [76,152]. Some strategies have been assessed to interfere with CSCs in preclinical models and patients, including inhibition of key CSC signaling pathways, viral therapy, awakening quiescent CSCs, and immunotherapy [144]. Currently, some anti-cancer drugs for targeting stemness, such as bedaquiline, have been approved for cancer therapy [153]. In addition, accumulating evidence has also revealed the promising clinical applications of circRNAs in CSC-targeted treatment, including functioning as new biomarkers, acting as vaccines, and bypassing the therapeutic resistance of CSCs [146]. For example, hg19_circ_0005033 promotes proliferation, migration, invasion, and chemotherapy resistance of laryngeal CSCs, acting as potential biomarkers for laryngeal CSCs diagnosis and therapy [154]. CircGprc5a could encode a peptide, which can bind with Gprc5a protein, a protein is highly expressed in bladder CSCs, suggesting that this peptide might function as a new surface antigen in bladder CSC vaccines [78]. Quiescence, a property that keeps a cell in a nondividing state (G_0_ phase) but allows the cell to re-enter the cell cycle at a later time, contributing to the therapeutic resistance of CSCs [146]. Currently, many functional studies have shown that circRNAs played roles in stem cell quiescence. For instance, circLMO7 can increase the number of myoblasts (a kind of unipotent stem cell) in the S-phase of the cell cycle and decrease the proportion of cells in the G_0_/G_1_ phase by sequestering miR-378a-3p [155]. Hence, circRNAs may be a new target and can provide new insights and directions to treat cancer and other diseases [146]. Collectively, the aforementioned studies indicate that the molecular mechanisms of circRNAs in CSCs are diverse, including modulating transcription, encoding peptides, braking mRNA, and sequestering miRNAs. Although existing studies suggest that circRNAs exert important effects on the regulation and progression of cancers and CSCs, research concerning the modulatory functions of circRNAs on CSCs is still in its infancy, and many issues still need to be further explored.

## circRNAs-mediated common pathways in stem cells

**Figure 4:**
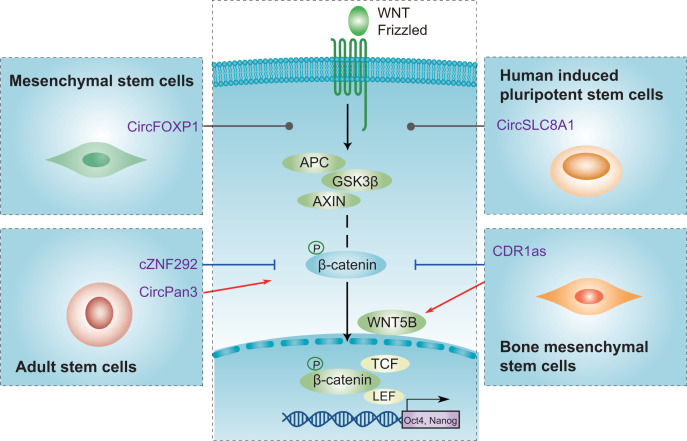
Emerging commonalities of circRNA-mediated signaling pathways When the typical Wnt signaling pathway is activated, β-catenin is secreted from the GSK3β-AXIN-APC complex. Then, β-catenin translocates to the nucleus and drives stemness. Certain circRNAs exert promotive (red) or suppressive (blue) roles by targeting different components of the Wnt pathway. Additionally, circRNAs can mediate the non-canonical Wnt signaling pathway to restrain stemness by up-regulating WNT5B expression (e.g., CDR1as). Abbreviations: hiPSC, human induced pluripotent stem cell; hiPSC-CM, human induced pluripotent stem cell cardiomyocyte.

Current evidence has suggested that circRNAs could be involved in the biology of stem cells by targeting the stemness-related pathway, such as the Wnt/β-catenin signaling pathway, and the downstream-related stemness genes, OCT4 and NANOG, to play a regulatory role (Figure 4). For example, circPan3 activates the β-catenin pathway and thus leads to the maintenance of Lgr5 ISCs [54]. However, cZNF292 has been shown to suppress the Wnt/β-catenin signaling pathway and increase OGD/R-induced NSCs injury [115]. In contrast, CDR1as facilitates WNT5B and alleviates β-catenin, thus leading to decreased osteogenic differentiation and increased adipogenic differentiation of BMSCs [62]. Additionally, circFOXP1 has been shown to orchestrate the non-canonical Wnt pathway and promote the proliferation and differentiation of MSCs [59]. HiPSCs derived from fibroblasts differentiate to hiPSC-CMs by modulating Wnt signaling, expressing diverse circRNAs, including circSLC8A1 [44]. Furthermore, certain circRNAs also regulate different stem cell types by influencing different pathways, including ERK, EGFR, and BMPs signaling pathways [48,59,69,115]. circRNAs participate in MSCs and ASCs by affecting ERK signaling pathway [58,69]. CircFOXP1 contributes to EGFR pathways regulation in maintaining MSC identity and manipulation of differentiation [59]. CircPOMT1 and circMCM3AP might regulate hASCs osteogenic differentiation via BMPs signaling pathway [146]. In summary, circRNAs mechanistically orchestrate Wnt signaling and thus expand our understanding of circRNA-mediated molecular mechanisms involved in stemness-related pathways.

The Wnt pathway is an evolutionarily conserved signaling pathway that determines different phenotypes, including apoptosis inhibition, dedifferentiation, and self-renewal of stem cells, suggesting that the Wnt pathway acts as therapeutic modalities [156–158]. Agents targeting stemness-associated markers in ongoing clinical trials are emerging. For example, temozolomide combined with ncRNAs (e.g., miR-125b) dramatically induced apoptosis by regulating the Wnt/β-catenin signaling pathway in glioma stem cells [159]. The combination of circRNAs and drugs targeting the Wnt pathway is expected to be a potential therapeutic strategy in stem cell therapy. Several drugs targeting the Wnt signaling pathway have been approved for clinical use by the FDA, such as non-steroidal anti-inflammatory drugs, such as celecoxib, which can suppress β-catenin degradation and has demonstrated anti-neoplastic activity in CRC cells [160,161]. Currently, many drugs target the Wnt signaling pathway, including small molecules (e.g., PRI-724), antibodies (e.g., OMP-18R5 against multiple Fzds), and recombinant proteins, which are being tested in Phase I trials [160]. Taken together, by targeting common Wnt pathway-associated factors, the function of different stem cells can be regulated in pre-clinical and clinical models. Drugs targeting the Wnt signaling pathway by manipulation of circRNAs may be potential therapeutic candidates. The development of drugs that target these crossover networks, when applied in combinatorial treatment, can potentially improve the efficacy of the treatment to a very large extent [162].

## Potential clinical application of circRNAs in stem cells

**Figure 5:**
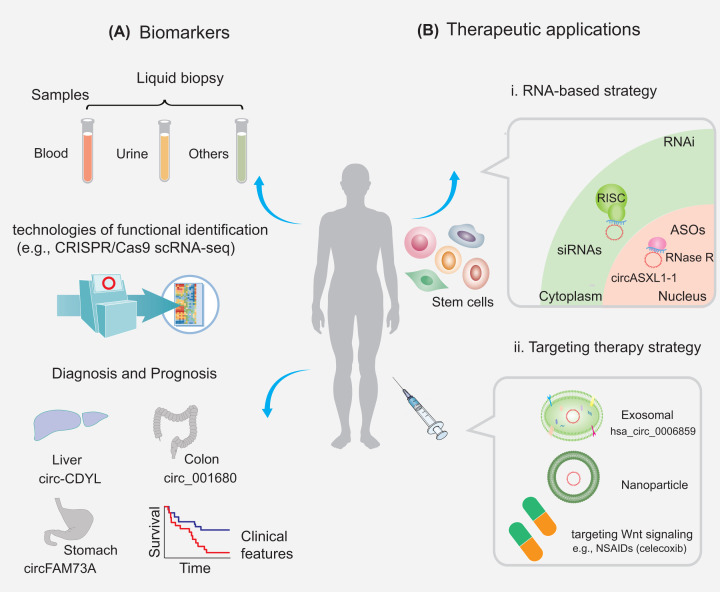
Potential clinical applications of circRNAs in stem cells Biomarker (**A**) and therapeutic (**B**) potential of circRNAs in stem cells. These circRNAs can be measured from different samples (e.g., blood and urine) and are potential biomarkers for the diagnosis and prognosis of diseases. RNAi and antisense oligonucleotide (ASO) agents can efficiently target circRNAs in the cytoplasm and nucleus, respectively, and circRNAs can be transferred by exosomes or nanoparticles. Abbreviations: RISC, RNA-induced silencing complex; RNase H, ribonuclease H; siRNA, small interfering RNA.

Emerging studies have shown that circRNAs act as key mediators of healthy and diseased states and may probably be utilized as prospective biomarkers for the diagnoses and prognoses of multiple diseases (Figure 5A). In addition, to be detected in body fluids such as gastric fluid, blood, and urine, circRNAs are relatively stable, conserved, and expressed in a cell type/developmental stage/tissue-specific pattern, thus enhancing the potential of circRNAs as treatment biomarkers for various diseases [163]. Notably, several promising techniques have emerged, such as single-cell RNA-sequencing, digital spatial profiling, and CRISPR/Cas9 [164–166]. With the advancements in the aforementioned promising technologies, numerous studies have been conducted to investigate the specific regulatory roles of circRNAs in an individual cell during the development and differentiation process of stem cells. To date, a small portion of circRNAs has been used as promising tools for diagnostic and prognostic biomarkers in certain diseases. For instance, hsa_circ_0006859 is a potential biomarker in human BMMSCs. Hsa_circ_0006859 differentiated osteopenia patients from healthy controls with an AUC of 0.913 (95% CI: 0.8617–0.9643, *P*<0.0001), 94.00% sensitivity, and 76.67% specificity. Hsa_circ_0006859 also significantly differentiated osteoporosis patients from healthy controls with an AUCof 0.8974 (95% CI: 0.8248–0.97, *P*<0.0001), 93.1% sensitivity, and 93.33% specificity [167]. Wei et al. identified that circ-CDYL (chromodomain Y-like), combined with hepatoma-derived GF and hypoxia-inducible factor asparagine hydroxylase, are both independent markers for discrimination of early stages of HCC with an odds ratio of 1.09 (95% confidence interval [CI]: 1.02–1.17) and 124.58 (95% CI: 13.26–1170.56), respectively. This study indicated that circ-CDYL contributed to the properties of epithelial cell adhesion molecule-positive liver TICs [168]. In addition, certain circRNAs (such as circ_001680, circFAM73A, and circRNA_103809) are highly expressed in distinct CSCs, suggesting that they act as novel biomarkers with promising diagnosis and prognosis significance [84,169,170]. To date, there are few biomarkers directly related to circRNAs and stem cells, and hence it can be a topic for future research related to targeted therapy.

The development of circRNA-targeted cancer treatment holds promise in the near future [171]. Future translational studies or clinical trials are warranted to develop circRNA-based therapeutics, which may eventually improve the prognosis of cancer patients via decreasing drug resistance. The development of antisense oligonucleotides (ASOs) to suppress the function of miRNAs was founded on the evidence that miRNAs govern their targets via base pair complementation [172]. Currently, RNA-based therapeutic approaches mainly include RNAi and ASOs, which can be designed to target a large and heterogeneous class of transcripts (Figure 5B) [173,174]. Loading of small interfering RNA or short hairpin RNA within stem cell-derived extracellular vesicles provides a safety advantage over the usage of stem cells themselves [175]. Jadhav et al. demonstrated that specific depletion of circASXL1-1 was accomplished employing ASO against the circASXL1-1 back-splice junction [105]. However, there are no clinical applications for targeting circRNAs. Some clinical trials with drugs based on ncRNAs, including miRNAs, with therapies that either up- or down-regulate the target miRNA, such as the first RNAi drug Onpattro, and the clinical success of the RNA-targeting oligonucleotide drug Spinraza, have been initiated for cancer treatment [26].

In circRNA-based treatment strategies, commonly used delivery systems targeting circRNAs include exosomes and nanoparticles (NPs) (Figure 5B) [176,177]. For example, CD133-derived exosomal circ-ABCC1 can regulate cell stemness and metastasis in CRC [178]. In addition, exosomes derived from stem cells carrying circRNAs could regulate new cell death patterns. For instance, UMSC-Exo could prevent pyroptosis and ischemic injury by releasing circHIPK3 [72]. Other researchers have reported that different stem cell-derived exosomal circRNAs could exert regulatory roles in liver fibrosis or diabetic ulcers [73,74]. Therefore, exosomes derived from stem cells, such as MSCs, are promising therapeutic cargoes for diverse diseases. Recent advances also show the possibility of targeting endogenous stem cells using NPs conjugated with specific biomolecules [179]. In addition, preclinical studies using NPs have demonstrated the feasibility of targeting chemo-resistant cancer cells, such as CSCs, to reverse clinical chemoresistance [180]. Increasing evidence has also indicated that NPs can track MSCs and manipulate MSCs functions [181]. NP delivery of synthetic oligonucleotides targeting oncogenic circRNAs, synthetic tumor suppressor circRNAs, and injection with natural agents for the modulation of circRNAs have been investigated as proof-of-concept in the treatment of various cancers [182]. Therefore, future research development of multifunctional NPs may open a new field for the application of targeting circRNAs in stem cell therapy.

## Conclusions and perspectives

In this review, we summarized some putative circRNAs in different stem cells, including ESCs, iPSCs, ASCs, MSCs, and CSCs. However, our understanding of the functions and mechanisms of action of diverse circRNAs in stem cells remains limited. Nevertheless, in the past decades, mounting evidence shows the significant progress that has been made in our understanding of circRNAs in stem cells at the epigenetic, transcriptional, and post-transcriptional regulatory levels. These findings driven by the implementation of new technologies, such as single-cell RNA-sequencing technology, have provided further insights into the evolution and heterogeneity of human diseases [183]. In term of CSCs, drug resistance is considered as a major challenge in cancer therapy. This scenario could be greatly improved because the advent of CRISPR-Cas9 screening technologies has greatly accelerated cancer research in many aspects, including robust site-specific gene editing, generation of animal cancer models, and functional genetic screening. The potential role of CRISPR-Cas9-based gene editing has received a lot of attention and has become a critical tool in the development of cancer therapeutics [184,185]. Therefore, with the development of these new technologies, targeting the cancer cell stemness-mediated pathways (that is, the Wnt signaling pathway) or identifying biomarkers is a crucial strategy to overcome drug resistance, thus improving the treatment outcome of cancer patients. Given their involvement in multiple mechanisms, the study of circRNAs will be key to improving our understanding of cancer biology.

Despite some progress of circRNAs have been made, the field is yet to conquer certain challenges and limitations. Current studies have shown the application potential of circRNAs as biomarkers in diverse cancer types; however, their diagnostic accuracy has yet to be validated through well-designed clinical trials [146]. To further accelerate this research field and the translation of basic findings in circRNAs into clinical settings, a series of crucial questions remain to be fully elucidated: (i) What are the suitable *in vivo* models to verify the *in vitro* findings on the functions of circRNAs in various stem cells? (ii) How to effectively avoid off-target effect when targeting circRNAs has been utilized as a novel therapeutic strategy? (iii) How to design good clinical trials to verify the diagnostic and prognostic potential of circRNAs as biomarkers for disease management? With rapid progress in the expanding regulatory mechanisms and cellular functions of circRNAs in stem cells, circRNAs are promising biomarkers and validated as therapeutic targets for stem cell-related diseases to improve treatment outcomes.

## Abbreviations

ADSC: adipose-derived stem cell
ALP: alkaline phosphatase
ASC: adult stem cell
ASO: antisense oligonucleotide
BMMSC: bone marrow mesenchymal stem cell
BMP: bone morphogenetic protein
BMSC: bone mesenchymal stem cell
CDR1as: cerebellar degeneration-related protein 1 transcript
cGAMP: cyclic GMP-AMP
cGAS: cyclic GMP-AMP (cGAMP) synthase
circRNA: circular RNA
CRC: colorectal cancer
CSC: cancer stem cell
EBVaGC: EBV-associated gastric cancer
EGFR: epidermal growth factor receptor
EpSC: epidermal stem cell
ERK: extracellular signal-regulated kinase
ESC: embryonic stem cell
hASC: human adipose-derived stem cell
HCC: hepatocellular carcinoma
hESC: human embryonic stem cell
hiPS: human induced pluripotent stem
HSC: hematopoietic stem cell
hucMSC: human umbilical cord-derived MSC
IFN: interferon
iPS: induced pluripotent stem
ISC: intestinal stem cell
LPS: lipopolysaccharide
miRNA: microRNA
MSC: mesenchymal stem cell
ncRNA: non-coding RNA
NELL-1: Nel-like molecule, type 1
NP: nanoparticle
NSC: neural stem cell
OGD/R: oxygen-glucose deprivation/reperfusion
ONFH: osteonecrosis of the femoral head
PDLSC: lipopolysaccharide-treated periodontal ligament stem cell
PTBP1: polypyrimidine tract-binding protein 1
RBP: RNA-binding protein
SREBP1: sterol-regulatory element-binding protein
SSC: spermatogonial stem cell
UMSC-Exo: umbilical cord mesenchymal stem cell-derived exosome
